# Long-term GreenFeed monitoring of enteric methane emissions in lactating buffaloes

**DOI:** 10.3389/fvets.2026.1946404

**Published:** 2026-08-31

**Authors:** Chiara Rossi, Giampiero Grossi, Nicola Lacetera, Andrea Vitali

**Affiliations:** Department of Agriculture and Forestry Sciences, University of Tuscia, Viterbo, Italy

**Keywords:** emission phenotyping, *in vivo* measurement technique, lactation curve, precision livestock farming, respiratory gas exchange, ruminants, temperature–humidity index, thermal load

## Abstract

The GreenFeed (GF) system is widely regarded as the silver standard for measuring individual enteric methane (CH_4_) emissions; however, it has not previously been used in dairy buffaloes (*Bubalus bubalis*). This study aimed to perform long-term monitoring using the GF in lactating Mediterranean buffaloes to provide benchmark values for enteric CH_4_ and carbon dioxide (CO_2_) emissions, and CH_4_ emission intensity, together with a characterization of CH_4_ emission patterns and animal-related factors. Additionally, seasonal variation in enteric CH_4_ emissions was evaluated. The study was conducted on a commercial dairy buffalo farm in central Italy from June 2024 to December 2025. A GF unit was installed in the feed alley, and animals were trained to voluntarily visit the system using pelleted concentrate as bait. Fifty-four lactating buffaloes were enrolled, of which 37 voluntarily visited the GF at least once during the monitoring period. Individual milk yield, days in milk (DIM), and parity were obtained from test-day milk records. Buffaloes visited the GF 1.6 ± 0.9 times/d on average, with only six animals reaching the maximum number of daily visits. Mean daily CH_4_ and CO_2_ emissions were 337.0 ± 108.3 and 9,190.1 ± 1,543.3 g/d, respectively, and a linear positive relationship was found between the two gases. The CH_4_ intensity averaged 44.4 ± 19.6 g/kg of milk. Methane emissions changed throughout lactation, increasing from early lactation to approximately 120 DIM before gradually declining thereafter. A strong positive relationship was observed between milk yield and CH_4_ emissions. Finally, average CH_4_ production was greater during late winter and spring than during summer and autumn. In conclusion, these findings provide the first on-farm benchmark values for enteric CH_4_ and CO_2_ emissions in Mediterranean dairy buffaloes obtained using the GF system and highlight the importance of accounting for productive level, lactation stage, parity, and seasonal variation when characterizing enteric CH_4_ emissions in this species. Further studies involving larger buffalo populations across multiple commercial farms are warranted to validate these findings and support the development of robust, species-specific enteric CH_4_ emission factors.

## 1 Introduction

Enteric methane (CH_4_) emissions from ruminants are recognized as a major contributor to agricultural greenhouse gas (GHG) emissions, accounting for 71% of CH_4_ emissions from the agricultural sector (1). As livestock production systems face increasing pressure to improve their environmental sustainability, accurate quantification of enteric CH_4_ emissions has become essential for developing effective mitigation strategies, refining national GHG inventories, and supporting climate-related policies (2, 3). Among livestock species, buffaloes are recognized for their adaptability to challenging environmental conditions, supporting their potential role in climate-resilient livestock systems (4).

Despite being the second-largest contributors to global enteric CH_4_ emissions, *in vivo* CH_4_ production in buffaloes remains considerably less investigated than in other ruminants (5). This knowledge gap is particularly relevant for dairy buffaloes (*Bubalus bubalis*), which play an important economic role in several regions of the world (6). Mediterranean dairy buffaloes represent the predominant buffalo population in Europe, which is concentrated almost entirely in Italy and primarily oriented toward the production of high-value dairy products, particularly mozzarella cheese (7).

In recent years, some *in vivo* techniques have been applied to quantify enteric CH_4_ emissions in dairy buffaloes under farm conditions, ranging from animal-invasive approaches, such as the sulfur hexafluoride (SF_6_) tracer technique, to non-invasive methods designed to facilitate farm monitoring, including laser CH_4_ detectors and sniffer systems (8–10). Among the available on-farm measurement techniques, the GreenFeed (GF) is one of the most adopted methods for quantifying individual enteric CH_4_ emissions, especially due to its demonstrated reliability in measuring eructated gases (11, 12). Nevertheless, to the best of our knowledge, its application in dairy buffaloes has not yet been reported.

*In vivo* studies on Mediterranean dairy buffaloes indicate that CH_4_ production increased from early to mid-lactation before declining and was higher in multiparous than primiparous animals (13). Additionally, seasonal effects have also been reported in growing Mediterranean buffaloes, where CH_4_ emissions were assessed during winter and summer seasons and exhibited significantly lower CH_4_ emissions and DMI during summer (14). However, individual observations of enteric emissions using high-accuracy measurement systems over extended periods remain largely unexplored in Mediterranean dairy buffaloes.

Therefore, this study aimed to provide the first long-term characterization of enteric emissions in Mediterranean dairy buffaloes using the GF system, regarded as a silver standard method for enteric emissions monitoring. Specifically, we investigated the variability in CH_4_ emissions associated with milk yield, parity, and DIM, and evaluated monthly variation and thermal conditions. The use of GF also enabled the provision of benchmark values for CH_4_ and CO_2_ daily production and the assessment of CH_4_ intensity (g CH_4_/kg milk), which reflects the amount of CH_4_ emitted per unit of milk produced.

## 2 Materials and methods

### 2.1 Animal management and data collection

The study was carried out on a commercial dairy buffalo farm located in the productive district of Amaseno (Frosinone, Italy) from June 2024 to December 2025. A group of 54 lactating buffaloes was enrolled in the monitoring trial. Animals were housed in a free-stall barn naturally ventilated through open sidewalls. Calves were separated from their dams immediately after birth and were not present before or during milking. Exogenous oxytocin was occasionally administered; however, its use was not recorded. Lactating buffaloes were milked twice daily through a conventional milking parlour at 5:00 a.m. and 3:00 p.m. Individual milk production, DIM, and parity were collected from the test-day milk records conducted by the Animal Breeders Association. At the beginning of the trial, the herd averaged 1.7 ± 0.9 parities, with a mean milk yield of 10.2 ± 2.7 kg/d. DIM averaged 133 ± 102, ranging from 14 to 601 during the monitoring period. Buffaloes were dried off according to the farm reproductive management, based on the expected calving date. However, lactation length was variable and could be extended in animals with delayed conception or managed under out-of-season strategies, resulting in lactations exceeding 600 DIM in some individuals. Animals were fed a total mixed ration (TMR) distributed twice daily at 6 a.m. and 4 p.m. In addition to the TMR, animals were provided with a daily fixed amount of 0.6 kg/head (0.5 kg as dry matter) of a commercial pelleted concentrate during milking sessions. Average administered diet composition and related chemical composition are indicated in Tables 1, 2, respectively.

**Table 1:** Ingredients of the average diet fed to lactating buffaloes during the monitoring trial, as dry matter (DM).

Administered diet	Amount (kg DM/head/day)
**TMR composition**	
*Forages*	
Mixed grass hay	4.8 ± 0.03
Maize silage	3.2 ± 0.4
Fresh grass	0.5 ± 0.2
*Concentrates*	
Wheat middlings	1.5 ± 0.01
Brewers	0.6 ± 0.8
Corn meal	2.9 ± 1.3
Soybean meal	1.2 ± 0.4
Fava bean	0.7 ± 0.3
Molasses	0.7 ± 0.0
Vitamin and minerals	0.2 ± 0.03
**Commercial concentrate^a^**	**0.5**
Total	16.8 ± 0.9

a Concentrate provided during milking sessions. Ingredients: wheat bran, wheat meal, glutinous corn flour, cane molasses, soybean meal (roasted and dehulled), sodium bicarbonate, calcium carbonate, sunflower meal (dehulled), yeast products (*Saccharomyces cerevisiae*), dried algae (*Chlorella vulgaris*).

**Table 2:** Chemical composition of the TMR and commercial concentrate included in the diet of lactating buffaloes.

Parameter^a^	TMR	Commercial concentrate
Dry matter, % as fed	54.3	90.0
Crude protein, % DM	15.2	14.6
Crude fat, % DM	2.3	3.7
Crude fiber, % DM	19.3	8.3
Starch, % DM	23.0	–
NDF, % DM	39.3	–
ADF, % DM	20.3	–
ADL, % DM	3.1	–
Ash, % DM	6.9	7.2
Sodium, % DM	0.1	0.5

a Chemical parameters of TMR were obtained from laboratory analyses performed by the company responsible for diet formulation and nutritional management at the buffalo farm. Chemical composition of the commercial concentrate was provided by the manufacturer.

### 2.2 GreenFeed monitoring setup

**Figure 1:**
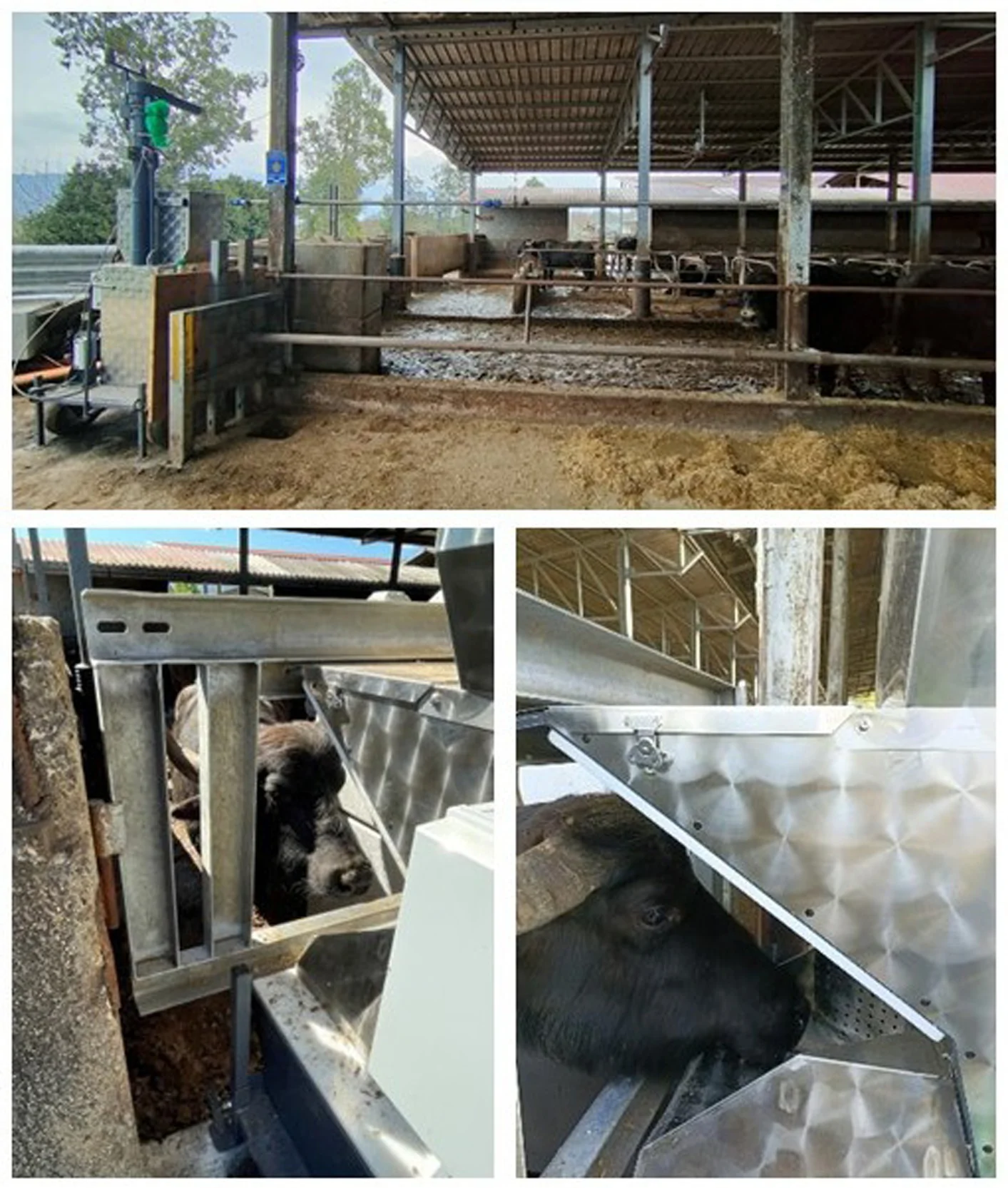
GreenFeed installation and animal visiting the unit at the dairy buffalo farm.

The GreenFeed (GF; C-Lock Inc., Rapid City, United States) unit shaped for animals with big horns was employed in the study to quantify the individual CH_4_ and CO_2_ production from lactating buffaloes’ exhaled breath. The unit was equipped with a semi-enclosed hood over a feeder where the air was continuously sampled at a flow rate of approximately 40 L/s. The unit was placed in the feed alley facing the group of lactating buffaloes. Animals were encouraged to visit the GF through the dispensing of pellet bait, which was set to provide a maximum of 8 feed drops per visit, consisting of 38 grams of pellet delivered every 30 s for a total of 4 min of visit duration. The pellet concentrate dispensed by the GF was the same type as that offered during the milking sessions; its ingredients and chemical composition are reported in the footnote to Table 1 and in Table 2, respectively. The drop mechanism allowed buffaloes to maintain a suitable head position during measurements, ensuring accurate gas detection. The dispensed bait concentrate was assumed to be completely consumed. Figure 1 shows the GF positioned in the barn and animals approaching the unit.

The GF was equipped with a radiofrequency identification (RFID) reader to identify ear-tagged animals, thereby enforcing a minimum interval between consecutive visits and enabling the assignment of emission measurements to individual animals. Additionally, the unit included a head position sensor to check the head proximity to the feeder. The mass flow of gases was estimated for each visit only when the animal’s head was correctly positioned and the visit lasted at least 2 min, in order to guarantee the recording of valid measurements (15). Buffaloes were allowed to visit the unit five times per day, having an overall supplementation of 1.52 kg of concentrate per head in addition to the TMR. CH_4_ and CO_2_ concentrations were recorded by NDIR sensors at 1-s intervals. Environmental (temperature, humidity, pressure, wind speed and direction) sensors were present on the unit to monitor ambient conditions. Before starting the monitoring trial, buffaloes were trained for 4 weeks to visit the GF and approach the unit voluntarily. Training sessions consisted of individually engaging buffaloes using a bin of pellet, and once the animal was near the GF feeder, manually dropping the bait from the unit.

As recommended by the manufacturer, gas calibrations were performed before and throughout the data collection period in accordance with the prescribed maintenance schedule. The GF calibration included a CO_2_ recovery test performed once a month, in which known CO_2_ standards were introduced into the unit to compare the injected quantity with the mass captured by the system. The GF procedure also included sensors calibration used to ensure that the reported concentration of gas sensors was correct. This calibration was conducted automatically every 6 days using a certified gas mixture to set both the baseline readings and the span with known concentrations of gases (3.5% CH_4_, 11.5% CO_2_, 0.04% H_2_, balance N_2_). Animal training and calibration procedures were accomplished according to Hristov et al. (16). The maintenance schedule of the GF unit also required the cleaning of the particulate air filter and head position sensor at least once per week. The GF unit underwent extraordinary maintenance only from October to November 2024, during which data collection was temporarily suspended.

Raw data cleaning and background gas correction were performed by C-Lock Inc. The processed data obtained from the GF consisted of the individual average emissions of CH_4_ and CO_2_, as grams, with a data aggregation per visit and day. Specifically, the average gas production per visit was calculated by summing the mass flow calculated for each second during a visit and dividing by the visit duration, after which the values were extrapolated to a 24 h period. Subsequently, the daily gas production for each animal was calculated by averaging the mass flow values obtained from valid visits per day (12).

### 2.3 Data management

Since individual milk yield data were recorded monthly through test-day milk records, daily milk production was estimated at the individual level using the Wilmink lactation model (17). This model is recommended by the International Committee for Animal Recording (ICAR) and has been applied to buffalo lactation curves, demonstrating good predictive performance (18–20).

The Wilmink function was defined as:


$$y(t)=a+b\cdot t+c\cdot e^{-k\cdot t}$$

where *y*(*t*) is the predicted daily milk yield (kg/day) at day in milk (*t*), (*a*) represents the level of milk production, (*b*) characterizes the increase in milk yield toward peak lactation, and (*c*) describes the decline in milk yield after peak lactation. Unlike the original Wilmink model, where the exponential constant (*k*) was fixed at 0.05 for dairy cattle, a study on Mediterranean buffaloes has estimated the *k* parameter specifically for this species [i.e., *k* = 0.169; (21)], which was adopted in the present study. The Wilmink parameters (*a*, *b*, and *c*) were estimated separately for each animal and lactation instead of relying on herd-average values. To estimate daily individual milk yield, the model was fitted using the monthly test-day milk records available for each single animal. This approach anchored the lactation curve to the individual test-day observations, enabling prediction of daily milk yield from the animal-specific lactation trajectory.

Before to proceed with statistical analysis, individual daily GF records of enteric CH_4_ and CO_2_ emissions, and the number of visits were associated with animal-specific characteristics, including parity, DIM, and the predicted daily milk yield for the corresponding day. Additionally, temperature and relative humidity data continuously recorded by the GF sensors installed on the feeding station were used to calculate the temperature–humidity index (THI) according to the equation described by Segnalini et al. (22). The resulting monthly THI values were then associated with the corresponding herd-level enteric CH_4_ emissions.

### 2.4 Statistical analysis

Statistical analysis was performed using Statistica 10 (Statsoft Inc., Tulsa, Oklahoma, United States). Individual data of enteric CH_4_ emissions and milk yield were analyzed using a mixed model, including parity (1, 2, 3+) as a fixed effect, individual daily GF pellet intake as a covariate, and animal as the repeated subject. Similarly, to evaluate monthly seasonal variation, daily CH_4_ emissions were analyzed using a mixed model, with month included as a fixed effect, individual daily GF pellet intake as a covariate, and animal as the repeated subject. When significant effects were detected, the Tukey test as post-hoc was performed. Significance was declared at *p* value < 0.05.

To describe the pattern of enteric CH_4_ emissions throughout lactation, DIM was grouped into 30-day intervals and the mean CH_4_ production was calculated for each class. The relationship between lactation stage and CH_4_ production was described by fitting a third-order polynomial regression to the class means. Similarly, to evaluate the relationship between milk yield and CH_4_ production, milk yield was grouped into 1-kg intervals, and the mean CH_4_ production was calculated for each class before fitting a linear regression model. Finally, the association between enteric CH_4_ and CO_2_ emissions was assessed by fitting a linear regression model. For all models, the regression equation and the coefficient of determination (*R*^2^) were reported to evaluate the goodness of the fit. For linear regression analyses, the statistical significance of the regression was assessed and the corresponding *p*-value was reported.

## 3 Results

### 3.1 Enteric emissions monitoring

**Figure 2:**
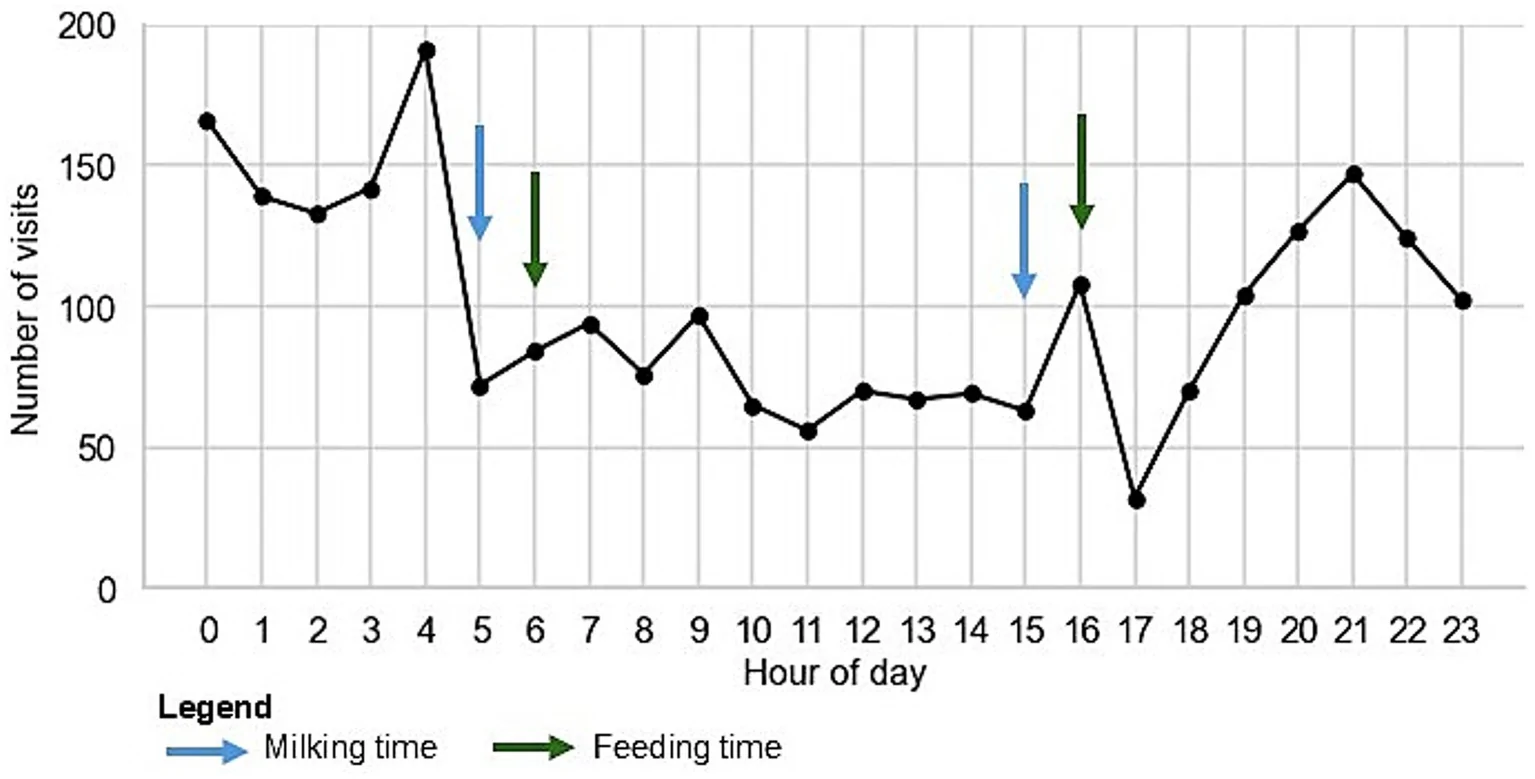
Daily distribution of GreenFeed visits throughout the monitoring period. Dots represent the cumulative number of visits recorded during each hour of the day.

Fifty-four lactating buffaloes were ear-tagged for GF detection, but only thirty-seven (68.5%) visited the unit at least once during the monitoring trial. On average, animals performed 1.6 ± 0.9 valid visits per day, with only six individuals reaching the maximum number of daily visits. The average daily pellet intake dispensed by the GF was 0.49 ± 0.27 kg/head, ranging from 0.30 to 1.52 kg/head per day. This variation in pellet intake resulted in an estimated total DMI ranging from 17.07 to 18.17 kg/head per day. Based on the average pellet intake, total nutrient intake was calculated by combining the chemical composition of the TMR and commercial concentrate, and it is reported in Supplementary Table S1. The cumulative distribution of GF visits recorded over the entire monitoring period varied across hours of the day (Figure 2). The highest visit frequencies were observed during the night and early morning, with a maximum at 04:00, followed by a decline corresponding to the morning milking time. During daytime hours, visit frequency peaked at 16:00 and reached its lowest value at 17:00. During the evening, the number of visits increased until reaching another peak at 21:00 before declining thereafter.

**Figure 3:**
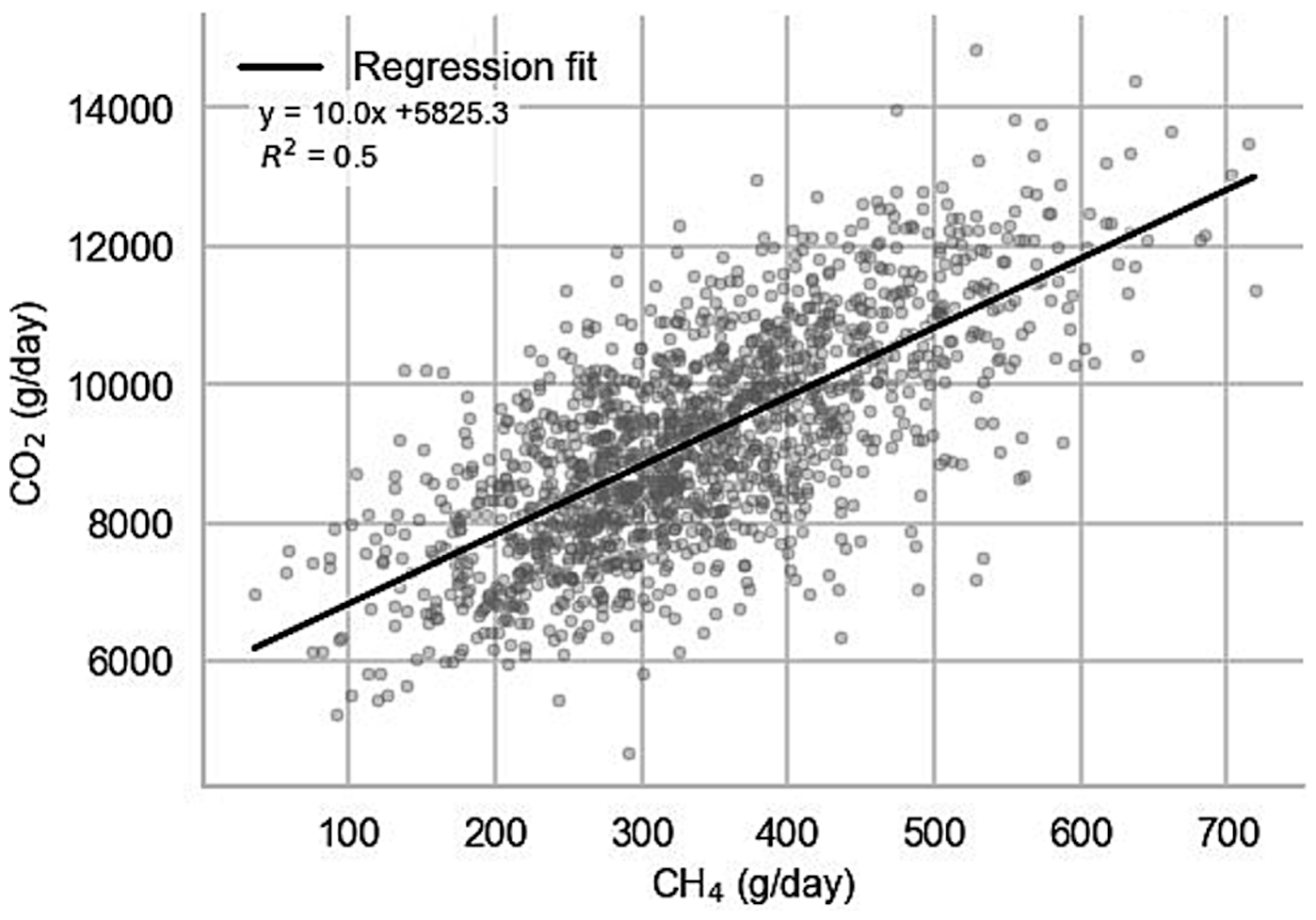
Linear regression between enteric CH_4_ and CO_2_ emissions monitored in lactating buffaloes by GreenFeed, considering all individual visits recorded throughout the monitoring period.

The results obtained from the long-term GF monitoring showed that average individual enteric CH_4_ and CO_2_ emissions of lactating buffaloes were 337.0 ± 108.3 and 9,190.1 ± 1,543.3 g/d, respectively, whereas CH_4_ emission intensity averaged 44.4 ± 19.6 g/kg of milk. Figure 3 illustrates the relationship between individual enteric CH_4_ and CO_2_ emissions recorded during the monitoring period. A significant positive linear association was observed across the records (*p* < 0.001). Linear regression analysis yielded the equation:


y=10.0⋅x+5,825.3

with a coefficient of determination (*R*^2^) of 0.50.

### 3.2 Effects of milk yield, lactation stage and parity on enteric CH_4_ emissions

The mixed-model analysis revealed a significant effect of parity class on both enteric CH_4_ emissions (*p* < 0.01) and milk production (*p* < 0.05). In contrast, daily GF pellet intake did not significantly affect either individual CH_4_ emissions or milk production. Table 3 presents enteric CH_4_ emissions and milk production according to parity classes (1, 2, and 3+). Data are reported as estimated means ± standard error (SE) derived from the mixed model, together with pairwise comparisons and corresponding *p*-values obtained using Tukey’s *post hoc* test. Specifically, primiparous buffaloes produced significantly less enteric CH_4_ (322.2 ± 6.3 g/d) compared to multiparous animals in both parity 2 (364.2 ± 10.7 g/d; *p* < 0.001) and parity 3 + (344.6 ± 7.4 g/d; *p* < 0.01). No significant difference in CH_4_ emissions was observed between parity 2 and parity 3 + (*p* = 0.08). Daily milk production was also significantly lower in primiparous buffaloes (7.8 ± 0.1 kg/d) than in second-parity (8.5 ± 0.2 kg/d; *p* < 0.001) and older multiparous buffaloes (8.7 ± 0.1 kg/d; *p* < 0.001).

**Table 3:** Enteric CH_4_ production and milk yield according to parity classes (1, 2, 3+) in lactating buffaloes.

Item	Parity classes			*P*-value		
	1	2	3+	1 vs. 2	1 vs. 3+	2 vs.3+
CH_4_, g/d	322.2 ± 6.3	364.2 ± 10.7	344.6 ± 7.4	<0.001	<0.01	0.08
Milk, kg/d	7.8 ± 0.1	8.5 ± 0.2	8.7 ± 0.1	<0.001	<0.001	0.2

**Figure 4:**
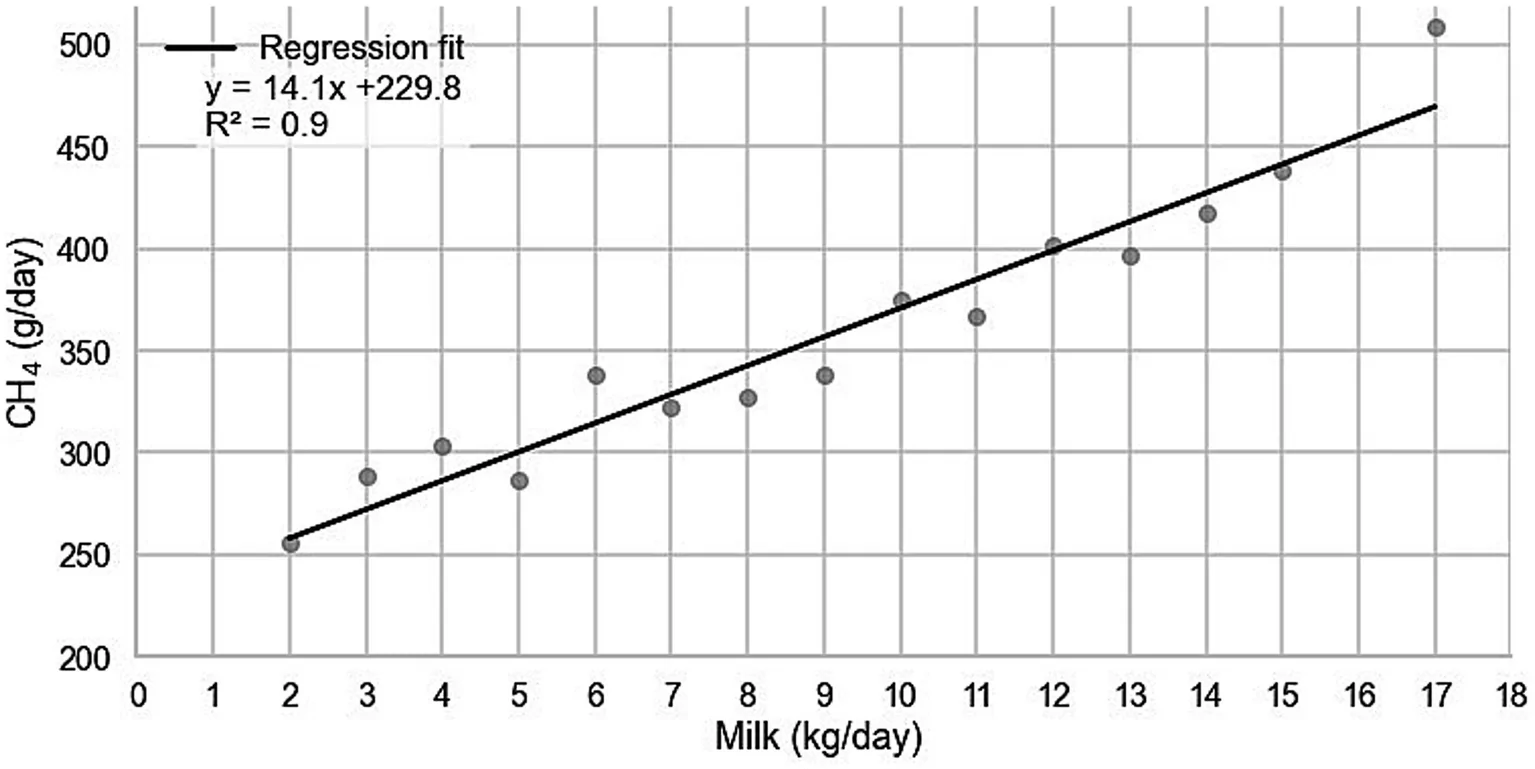
Linear regression between milk production and enteric CH_4_ emissions monitored in lactating buffaloes. Data points represent mean CH_4_ emissions for 1-kg milk yield intervals.

Figure 4 shows a significant positive linear relationship between milk yield and enteric CH_4_ emissions (*p* < 0.001). The linear regression describing CH_4_ production (*y*) pattern as a function of milk yield (*x*) was described by the equation:


y=14.1⋅x+229.8

The regression showed a coefficient of determination *R*^2^ = 0.90. According to the model, enteric CH_4_ emissions increase of 14.1 g/d for each kilogram of milk produced per day.

**Figure 5:**
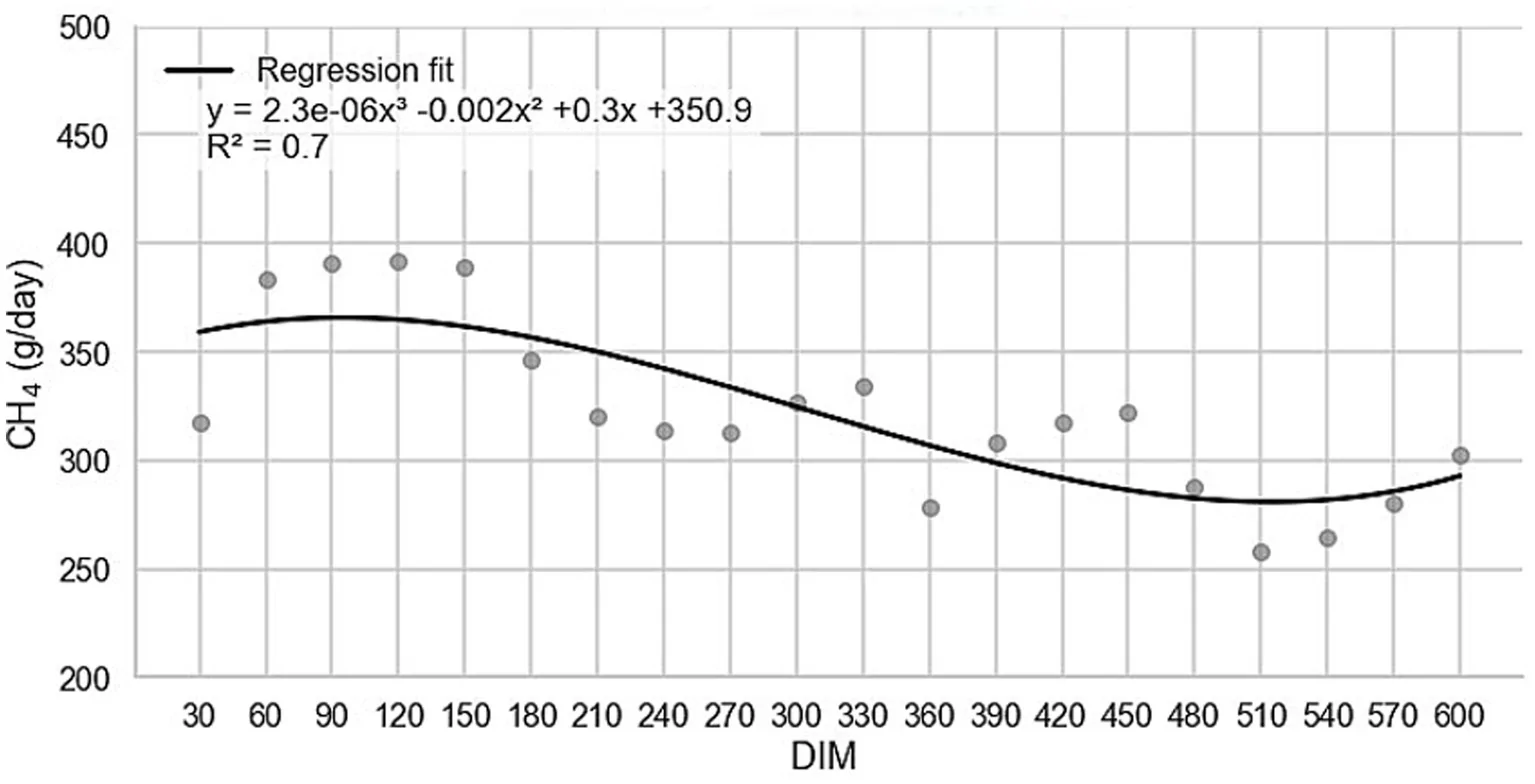
Third-order polynomial regression describing the relationship between days in milk (DIM) and daily enteric CH_4_ emissions in lactating buffaloes. Data points represent mean CH_4_ emissions for 30-day DIM intervals.

The relationship between lactation stage and daily enteric CH_4_ production was analyzed across 30-day DIM intervals using a third-order polynomial regression (*R*^2^ = 0.7; Figure 5). Daily CH_4_ production increased during early lactation (30 to 120 DIM), reaching values approaching 400 g/d between 90 and 120 DIM. Thereafter, CH_4_ production decreased across mid- and late lactation, reaching a minimum value of 258 g CH_4_/d. The polynomial regression equation describing CH_4_ production (*y*) pattern as a function of DIM (*x*) is expressed as:


$$y=2.3\cdot e^{-0.6x^{3}}-0.002x^{2}+0.3x+350.9$$

Supplementary Table S2 presents the number of individuals, GF visits and milk yield for each 30-day DIM interval.

### 3.3 Monthly enteric CH_4_ emission pattern in relation to climate conditions

**Figure 6:**
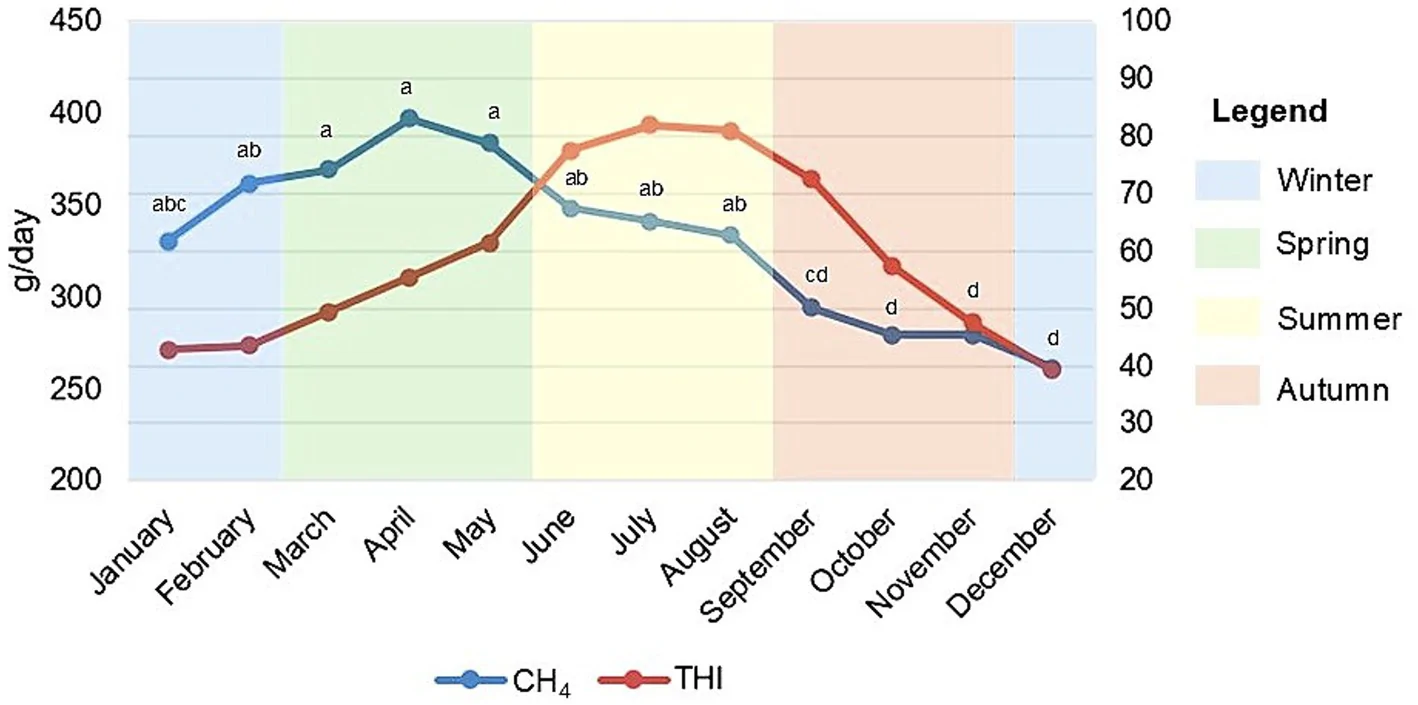
Monthly variation of herd-level enteric CH_4_ emissions and temperature–humidity index (THI) recorded throughout the monitoring period. Seasons are highlighted with color shaded bands. Different letters indicate significant differences among monthly mean CH_4_ emissions. Differences among months were evaluated using Tukey’s *post hoc* test.

The mixed-model analysis revealed a significant effect of month on enteric CH_4_ emissions (*p* < 0.001), whereas the individual daily GF pellet intake did not exhibit a significant effect. The Tukey *post hoc* test indicated significant differences among months (*p* < 0.05), as shown in Figure 6. Emissions increased from 330.1 g/d in January to 397.0 g/d in April and subsequently decreased, reaching the lowest value of 261.6 g/d in December (Figure 6). The highest CH_4_ emissions were observed in March, April, and May, whereas December, October, and November showed the lowest values. The THI, which reflects thermal conditions and associated heat stress in the barn, increased from winter to summer, reaching its highest values in July and August, and decreased thereafter toward the end of the year. Overall, enteric CH_4_ emissions showed a declining trend with increasing THI.

## 4 Discussion

The use of GF in the commercial dairy buffalo farm revealed some limitations in terms of animal willingness to visit the unit, as reflected by the low average number of individual daily visits compared with the five potentially available throughout the day. At the beginning, animals require training to consistently approach the GF unit voluntarily, but later on, their engagement may vary over time and be influenced by herd hierarchies, potentially leading to uneven distribution of animal visits to the unit. In the present study, buffaloes performed an average of 1.6 ± 0.9 GF visits per day, which was lower than the frequencies reported for dairy cows under both confined and grazing conditions, ranging from 4.3 to 2.5 times per day (23, 24). Despite the lower visitation frequency, the number of enrolled animals (54) was consistent with previous recommendations indicating that a single GF unit can successfully monitor up to 50–60 dairy cows under confined conditions (25). Therefore, the low frequency of GF visits was not due to a limited number of animals engaged with the unit, whereas the placement of the GF likely contributed to this limitation. Methodological recommendations suggest positioning the unit in highly accessible areas, such as central locations within the barn or close to water stations. Additionally, it is preferable to equip the unit with a rear gate to ensure single-animal access and minimize social interference during concentrate consumption (25). Alvarez et al. (26) positioned the GF unit according to similar recommendations on a commercial dairy cow farm and reported successful animal visitation. However, their study involved cows already accustomed to automated systems, including a robotic milking system, three concentrate feeders, and two GF units. Therefore, their previous experience with automated equipment may have facilitated the animals’ voluntary engagement with the GF units.

In the enrolled commercial farm, the installation site was constrained by the available space in the barn; therefore, the GF unit could only be positioned in a location that was not along a natural passage route and without an automatic rear gate. These conditions likely reduced the frequency of voluntary visits. Additionally, the farm was not equipped with automated milking or feeding systems; therefore, the animals had no previous experience interacting with automated equipment, which may have further limited their voluntary engagement with the GF unit. Potential species-specific behavioral differences between dairy cows and buffaloes in their adaptation time and response to GF cannot be excluded, as this is the first application of a GF unit in buffaloes and current recommendations for large ruminants are based on cattle. Therefore, future GF installations in dairy buffalo farms should prioritize accessibility, integration with the animals’ natural movement patterns, and controlled single-animal access to minimize social competition and improve visitation frequency to the unit. Nevertheless, the extended monitoring period ensured that each animal performed at least 8 visits over multiple days and at different times of the day. This sampling approach is consistent with recent evidence showing that 8 randomly distributed GF spot samples per animal are sufficient to provide reliable estimates of average CH_4_ and CO_2_ emissions under commercial dairy conditions (40). Additionally, the long-term monitoring allowed for the characterization of the hourly pattern of animal visits to the GF throughout the day, which has been broadly demonstrated in dairy and growing cattle to be strongly influenced by milking and feeding schedules (27–29). Similar to other GF studies, animals exhibited greater visitation activity during the night than during daytime periods, likely reflecting the combined effect of animal behavior and farm management routines (30, 31).

The long-term monitoring trial provided benchmark values for enteric CH_4_ and CO_2_ emissions in Mediterranean lactating buffaloes using the GF system. The average daily CH_4_ emissions were in line with those reported using the SF_6_ tracer technique by Hossain et al. (41) and Singla et al. (10), who measured daily CH_4_ emissions of 256 and 260 g/d in Murrah and Surti × Mehsana lactating buffaloes, respectively. The 21–23% higher CH_4_ emissions observed in the present study may be attributable to the greater body weight of Mediterranean buffaloes, estimated around 650 kg for multiparous and 570 kg for primiparous animals (32), compared with the 503–558 kg reported for the breeds evaluated in the previous studies. In contrast, Petrocchi Jasinski et al. (8) monitored lactating Mediterranean buffaloes using a laser CH_4_ detector and reported average enteric CH_4_ emissions approximately 17% higher than those obtained with the GF. Nevertheless, it is important to note that all previous studies quantifying enteric CH_4_ emissions refer to short-term monitoring periods, ranging from 40 to 120 days; consequently, the reported emission levels may reflect specific environmental and management conditions rather than representative enteric CH_4_ production.

With regard to enteric CO_2_ emissions, available literature provides limited information from *in vivo* monitoring. However, in our previous work (9), we used the sniffer system to monitor lactating Mediterranean buffaloes on the same commercial dairy farm, and although the monitored animals were not the same individuals, the estimated CO_2_ emissions were consistent with those observed here. Moreover, the positive linear relationship between enteric CH_4_ and CO_2_ emissions agrees with previous findings obtained using respiration chambers, SF_6_ technique and GF on dairy cattle (28, 33). Enteric CH_4_ is produced by ruminal methanogenic archaea, which utilize hydrogen and CO_2_ generated during microbial fermentation, whereas exhaled CO_2_ originates from both ruminal fermentation and, predominantly, the animal’s oxidative metabolism (30, 34). Consequently, CO_2_ production reflects the animal’s metabolic activity and forms the physiological basis of the CH_4_:CO_2_ ratio method proposed by Madsen et al. (34), in which CO_2_ is used as an internal tracer to estimate enteric CH_4_ emissions. More recently, Bayat et al. (30) confirmed the positive relationship between CH_4_ and CO_2_ exchanges measured using both the GF and respiration chambers, and demonstrated that CO_2_ exchange is more closely associated with heat production than CH_4_ emissions. The differences in CH_4_ and CO_2_ biological origins are consistent with the moderate coefficient of determination observed in the present study (*R*^2^ = 0.50), indicating that a considerable proportion of the variability in CH_4_ and CO_2_ emissions remains independent.

The associations between enteric CH_4_ emissions and both DIM and parity observed in the present study are consistent with the findings of Meo Zilio et al. (13), who identified both factors as significant determinants of CH_4_ emissions in Mediterranean dairy buffaloes measured using a laser CH_4_ detector. The authors described a CH_4_ emission curve characterized by a peak followed by a progressive decline, a pattern that was also observed in the present study, suggesting to be superimposable to the buffalo lactation curve. These findings enforce the contrast with those reported in dairy cattle, where enteric CH_4_ emissions increase during early lactation and subsequently remain stable throughout the rest of lactation (35–37). It should be noted that in the present study the tracking of enteric CH_4_ emissions reaches up to 600 DIM, exceeding the standard 300 days of buffalo lactation length (38). This phenomenon reflects the occurrence of extended lactations within buffalo herds due to reproductive management challenges, such as delayed conception and the application of out-of-season calving strategies to align peak milk production with summer market demands for cheese production (39). For this reason, buffaloes with long calving intervals remain in the milking herd for extended periods, showing remarkable persistency. In agreement with Meo Zilio et al. (13), primiparous animals emitted less CH_4_ than multiparous buffaloes, confirming that parity should be considered when evaluating enteric CH_4_ production in this species. The lower CH_4_ emissions observed in primiparous buffaloes likely reflect their lower body weight and productive performance compared with multiparous animals, which may be attributable to a lower DMI. Nevertheless, no significant differences were detected between parity 2 and parity 3 + buffaloes, suggesting that once animals reach mature productive performance, parity per se may have only a limited influence on daily CH_4_ production.

The monthly variation in enteric CH_4_ emissions observed in the present study indicated a decrease as THI started to increase, corresponding to the late spring and summer season. A similar seasonal effect was reported by Lanzoni et al. (14), who indicated lower enteric CH_4_ emissions during summer compared with winter in Mediterranean buffaloes and attributed this response to heat stress-induced reduction in DMI. Nevertheless, the GF monitoring showed that the decline in CH_4_ production continued until December, when THI values were well below the threshold associated with heat stress. This suggests that a combination of factors, rather than heat stress alone, may have contributed to the seasonal variation in CH_4_ emissions. The hypothesis proposed by Meo Zilio et al. (13), who found a similar monthly pattern with peak CH_4_ emissions in April and the lowest point in January, highlights the potential role of photoperiod in influencing both the physiological status of animals and enteric CH_4_ emissions, particularly in sensitive species such as buffaloes. However, because photoperiod covaries with other seasonal factors, including forage quality, management practices, and stage of lactation, in the present study the relative contribution of each factor could not be disentangled. Further investigations under controlled conditions are therefore warranted to clarify whether the photoperiod effect may contribute to the variation in enteric CH_4_ emissions in Mediterranean buffaloes.

## 5 Study limitations and future research

The present study has some limitations that should be considered. The individual DMI and body weight were not directly measured. Consequently, the observed associations between enteric CH_4_ emissions and animal-related characteristics, such as milk yield, parity and DIM may be partially influenced by differences in feed intake and body weight. A further limitation relates to the variation in GF visit frequency among animals. The non-uniform temporal distribution of the visits throughout the day may have influenced the representation of the daily individual emission estimates. Additionally, it resulted in different amounts of bait concentrate consumed. To minimize this source of variation and its potential effect on CH_4_ emissions, daily pellet intake was included as a covariate in the statistical mixed models; however, it did not exhibit a significant effect on the CH_4_ emissions. Nevertheless, some residual effect associated with the temporal distribution of GF visits cannot be excluded. Another limitation refers to the sample size. The study was conducted on a single commercial dairy buffalo farm, and only 37 of the 54 enrolled animals visited the GF during the trial. Consequently, the number of animals and records across months, DIM and parity classes could not be standardized, as this would have required excluding additional observations. Future studies involving a larger number of dairy buffalo farms and a wider buffalo population would allow a more equal number and temporal distribution of GF visits, as well as a more balanced representation of animal characteristics for parity and stage of lactation. In addition, the inclusion of individual measurements of total DMI, body weight, milk composition, and actual bait consumption would help disentangle the effects of animal-related factors and feeding management on enteric CH_4_ emissions, thereby improving the robustness of enteric emission estimates.

## 6 Conclusion

Although the implementation of GF in the commercial dairy buffalo farm presented several challenges, the adoption of a long-term monitoring approach enabled the characterization of enteric CH_4_ and CO_2_ emissions under commercial farming conditions. The findings supported the hypothesis that enteric CH_4_ emissions varied according to animal-related factors, including milk yield, parity and stage of lactation, as well as according to monthly seasonal conditions. However, individual DMI may also have contributed to the observed variability. This study provides the first benchmark reference values of enteric CH_4_ and CO_2_ emissions obtained using the GF in dairy buffaloes and offers practical indications for future studies employing this technology in the species.

## Data Availability

The raw data supporting the conclusions of this article will be made available by the authors, without undue reservation.
