# Supplementary material for: Long-term GreenFeed monitoring of enteric methane emissions in lactating buffaloes
Source: Front Vet Sci. 2026 Aug 31;13:1946404. doi: 10.3389/fvets.2026.1946404 (PMC13572151; doi:10.3389/fvets.2026.1946404)
Supplement: Supplementary file 1 [file Table_1.DOCX]

| Table S1. Estimated daily total nutrient intake per head based on the average intake of total mixed ration (TMR) and commercial concentrate provided during milking sessions and GreenFeed visits. | |
| --- | --- |
| **Parameter** | **Total nutrient intake (kg/head/d)** |
| Dry matter TMR | 16.32 |
| Dry matter conc milking | 0.54 |
| Dry matter conc GreenFeed | 0.44 |
| Total dry matter | 17.28 |
| Crude protein | 2.62 |
| Crude fat | 0.41 |
| Crude fiber | 3.23 |
| Starch | 3.75 |
| NDF | 6.41 |
| ADF | 3.31 |
| ADL | 0.51 |
| Ash | 1.20 |
| Sodium | 0.02 |

| Table S2. DIM classes characteristics. | | | |
| --- | --- | --- | --- |
| **DIM class** | **Number of individuals^1^** | **GF visits** | **Milk yield** |
| 0-30 | 4 | 49 | 8.2±0.6 |
| 30-60 | 7 | 68 | 9.1±2.3 |
| 60-90 | 13 | 117 | 11.7±2.6 |
| 90-120 | 17 | 269 | 11.0±1.7 |
| 120-150 | 18 | 188 | 10.6±1.6 |
| 150-180 | 17 | 281 | 8.8±2.3 |
| 180-210 | 17 | 210 | 7.5±1.5 |
| 210-240 | 18 | 256 | 6.9±1.8 |
| 240-270 | 21 | 201 | 6.9±2.1 |
| 270-300 | 19 | 142 | 6.6±1.4 |
| 300-330 | 11 | 46 | 6.4±1.7 |
| 330-360 | 8 | 76 | 6.8±2.5 |
| 360-390 | 5 | 67 | 6.9±2.9 |
| 390-420 | 6 | 82 | 7.3±2.8 |
| 420-450 | 7 | 48 | 6.7±2.8 |
| 450-480 | 5 | 45 | 6.3±2.9 |
| 480-510 | 7 | 94 | 6.1±1.7 |
| 510-540 | 6 | 80 | 6.3±2.6 |
| 540-570 | 3 | 63 | 5.2±1.7 |
| 570-600 | 2 | 37 | 4.0±1.5 |
| ^1^Individual animals contributed to observations in multiple DIM classes. | | | |
